# Multiple moderator meta-analysis using the R-package Meta-CART

**DOI:** 10.3758/s13428-020-01360-0

**Published:** 2020-06-15

**Authors:** Xinru Li, Elise Dusseldorp, Xiaogang Su, Jacqueline J. Meulman

**Affiliations:** 1 Mathematical Institute, Leiden University, P.O. Box 9512, 2300 RA Leiden, The Netherlands; 2 Institute of Psychology, Leiden University, P.O. Box 9555, 2300 RB Leiden, The Netherlands; 3 Department of Mathematical Sciences, University of Texas at El Paso, Austin, TX 78712 USA; 4 Department of Statistics, Stanford University, Stanford, CA 94305 USA

**Keywords:** Meta-analysis, Heterogeneity, Interaction between moderators, CART, Fixed effect, Random effects, Computer software

## Abstract

In meta-analysis, heterogeneity often exists between studies. Knowledge about study features (i.e., moderators) that can explain the heterogeneity in effect sizes can be useful for researchers to assess the effectiveness of existing interventions and design new potentially effective interventions. When there are multiple moderators, they may amplify or attenuate each other’s effect on treatment effectiveness. However, in most meta-analysis studies, interaction effects are neglected due to the lack of appropriate methods. The method meta-CART was recently proposed to identify interactions between multiple moderators. The analysis result is a tree model in which the studies are partitioned into more homogeneous subgroups by combinations of moderators. This paper describes the R-package **metacart**, which provides user-friendly functions to conduct meta-CART analyses in R. This package can fit both fixed- and random-effects meta-CART, and can handle dichotomous, categorical, ordinal and continuous moderators. In addition, a new look ahead procedure is presented. The application of the package is illustrated step-by-step using diverse examples.

**Electronic supplementary material:**

The online version of this article (10.3758/s13428-020-01360-0) contains supplementary material, which is available to authorized users.

## Introduction

Methodology for synthesizing findings from multiple studies addressing the same research question has a long history (Hedges, 1981; Hedges & Olkin, 1985). The typical goals of meta-analysis are to estimate the overall effect size (i.e., a weighted average of study effect sizes), to quantity the heterogeneity in the study effect sizes, and to investigate the study characteristics that explain the heterogeneity (i.e., moderators). The relationship between moderators and the study effect sizes can be of high interest for behavioral scientists to evaluate existing interventions and to design new potentially effective interventions. In the meta-analysis framework, moderator analysis can be conducted with several stand-alone software programs such as **Comprehensive Meta-Analysis** (Borenstein et al., 2009), **Meta-Analyst** (Wallace et al., 2009), and the Cochrane Collaboration’s **RevMan** (Collaboration, 2014). Also, there are add-ins/macros/packages that can be used to conduct moderator analysis in many software languages such as **Stata** (StataCorp 2017, for details, see Sterne et al.,^2008^), **SAS** (Inc, 2002, for details, see Wang & Bushman, 1999; Arthur et al., 2001, and Sheu & Suzuki, 2001), **SPSS** (IBM Corp, 2013, for details, see Lipsey & Wilson, 2001), and **R** (Team, 2017), e.g., package **rmeta** by Lumley, 2012, **mvmeta** Gasparrini et al. (2012), and **metafor** by Viechtbauer (2010)). Most of these programs/add-ins are based on meta-regression, which is the most commonly used method for moderator analysis in meta-analysis.

In practice, behavioral scientific meta-analyses often have multiple moderators to be examined. For example, interventions to change health-related behavior generally include various behavior change techniques (BCTs), and researchers are interested in investigating the influence of BCTs on the effectiveness of interventions (see Michie et al.,^2009^). However, meta-regression has several limitations in such cases. First, when the number of included studies is small, meta-regression suffers from low statistical power to examine all moderators simultaneously (Tanner-Smith & Grant, 2018). Second, meta-regression has difficulties in exploring interaction effects among moderators since it requires moderators and their interactions to be specified beforehand. When there are no a priori hypotheses available, the number of all possible interaction terms are usually too large to be included in one model. The interaction effects, however, can provide valuable information to answer questions such like “do these intervention components amplify or attenuate each other’s effectiveness?” and “which combination of study characteristics results in the highest effectiveness?”.

To overcome the aforementioned limitations, tree-based models can be integrated into the framework of meta-analysis. Tree-based methods were introduced for the first time by Morgan and Sonquist (1963) in a method called automatic interaction detection (AID), and were fully developed in classification and regression trees (CART) by Breiman et al., (1985). Trees are good at dealing with many predictor variables that may interact, and produce results that can be easily interpreted. Tree-based methods have been used in the field of behavioral and medical sciences (for details, see Finch et al., 2011; Leach et al., 2016; Trujillano, Badia, Serviá, March, & Rodriguez-Pozo, 2009), but the idea of using trees in the meta-analysis framework is relatively new. For individual patient data (IPD) meta-analyses, Mistry et al., (2018) proposed a recursive partitioning method called IPD-SIDES that identifies patient subgroups by individual characteristics that may be related to the response to intervention. For aggregated data meta-analyses, a method called meta-CART was proposed to identify interaction effects among study-level characteristics (Dusseldorp et al., 2014; Li et al., 2017; 2019). This paper focuses on moderator analyses on aggregated meta-analysis by meta-CART. Compared to meta-regression, meta-CART has several advantages: first, it excels at dealing with interaction effects, and the interactions can be easily interpreted; second, it has automatic variable selection and does not require model selection; third, it is able to handle non-linear associations between moderators and effect size. Furthermore, since tree models are invariant to monotone transformation of predictors, meta-CART can keep the ordering information of ordinal moderators, whereas meta-regression usually codes ordinal variables the same as categorical variables. Li et al., (2019) showed via a simulation study that with a sufficiently large sample size (i.e., *n* ≥ 40 for simple interactions and *n* ≥ 80 for complex interactions), meta-CART has a good control of type I error rate (≤ 0.05), and can achieve satisfactory power and recovery rates (i.e., ≥ 0.80). The meta-CART method has been acknowledged as a potential alternative statistical method for meta-regression to understand the combined effects of moderators (O’Brien et al., 2015; Michie et al., 2015; Tipton et al., 2018), and it has been applied in several meta-analytic studies (e.g., Bull et al., 2018; van Genugten et al. 2018).

In the present paper, we introduce the R package **metacart**, which implements the meta-CART method. This package provides user-friendly functions to perform meta-CART analysis for various types of moderators (i.e., continuous, ordinal, and categorical variables), and includes various additional options such as tuning the pruning of the tree model, restricting the minimum number of studies in a subgroup, and so on. In addition, we developed a new option to apply a “look-ahead” strategy specifically focusing on interaction detection. This paper aims to provide a general overview of the capabilities of the **metacart** package for conducting multivariate moderator meta-analysis with R. “Meta-CART method” introduces the meta-CART method. “The metacart package” describes the main functions in the **metacart** package, and “Examples” illustrates their practical usage with examples of real meta-analyses. “Conclusions” contains concluding remarks.

## Meta-CART method

### The goal of meta-CART analysis

**Fig. 1:**
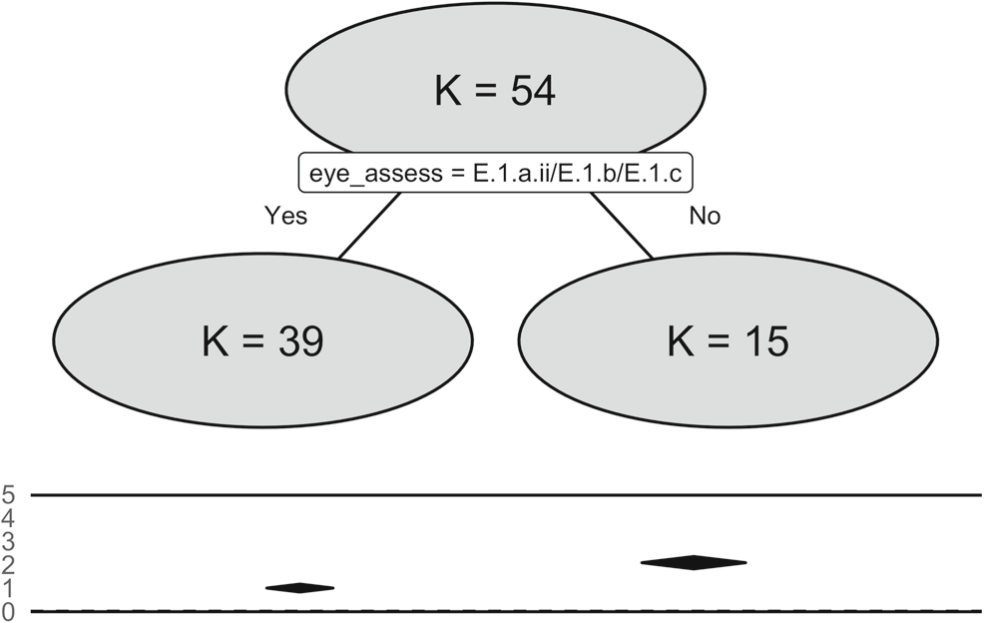
The meta-CART analysis results of 54 samples examining the influence of moderators on the association between handedness and eye-dominance. The figure shows the main effect of the method to assess eye-dominance, which partitions the samples in two subgroups. The *two solid lines* show the range of the effect sizes of all the studies. The *diamonds* between the solid lines present the 95% confidence intervals of the summary effect sizes

The underlying goal of meta-CART analysis is to identify subgroups defined by the moderators that can explain the heterogeneity in the study effect sizes. Appropriate data for meta-CART include an outcome variable of interest (i.e., study effect size), the within-study sample variance of the effect size, and the potential moderator variables that may influence the effect size. Depending on the type of study, there is a variety of different effect-size measures, including the odds ratio, the relative risk, the correlation coefficient, and (the standardized) mean difference. Meta-CART can deal with all these effect-size measures as long as all the studies use the same measure^1^. To identify influential moderators, meta-CART partitions the studies into subgroups that are more homogeneous with respect to their study effect sizes. The result is a tree model with the terminal nodes as the identified subgroups and the splitting variables as the influential moderators. Figure 1 shows an example of a tree model fitted by the package **metacart**. The root node (i.e., the node at the top) represents all the studies that are included in the analysis. From the root node, the studies are partitioned into two subgroups by applying a threshold on the values of a moderator. For example, the root node in Fig. 1 is partitioned into two child nodes based on the moderator “eye_assess”. This moderator is categorical, and the studies of which “eye_assess” equals to “E.1.a.ii”, “E.1.b”, or “E.1.c” fall into the left child node and the other studies fall into the right child node. If the moderator is an ordinal or a continuous variable, a binary question such as “is the value of the moderator smaller than the split point?” will be asked to introduce a split. Note that the moderators and the corresponding split points are automatically selected by the algorithm. How the moderators and split points are selected will be explained in “Meta-CART algorithm”.

The resulting subgroup memberships are defined by a hierarchically nested set of decision rules such as “does the intervention include certain treatment components?” or “is the average age of the patients smaller than 25?”. In the identified subgroups, the summary effect sizes and their confidence intervals are estimated. 2 The analysis results can be used by researchers to predict the effect size given the study characteristics, or to identify the combination of study characteristics that results in the highest/lowest effect size. Note that meta-CART has an exploratory nature, since the subgroups are not predefined but identified from the data. Thus, meta-CART should be used as a hypothesis-generating tool, and we recommend testing the identified moderators by confirmatory methodology in further studies.

### Meta-CART algorithm

There are two types of meta-CART algorithm: fixed-effect (FE) meta-CART, a partitioning algorithm that ignores the residual heterogeneity unexplained by the moderators, and random-effects (RE) meta-CART, a partitioning algorithm that takes into account the residual heterogeneity. As in standard meta-analysis, the choice between FE and RE assumptions should be based on a researcher’s prior belief; in other words, a researcher should decide on which assumption to use before analyzing the data. In general, if a researcher believes that given the study characteristics the variance of the study effect sizes is merely due to the sampling variance^3^, FE meta-CART can be chosen. If there is no a priori information about the residual heterogeneity, RE meta-CART is recommended. A more general discussion about FE model and RE model in meta-analysis can be found in Borenstein et al., (2010) and Schmidt et al., (2009).

In this section, we describe the FE and RE meta-CART algorithms, which underly the **metacart** package.

#### Fixed effect meta-CART

FE meta-CART splits the studies into more homogeneous subgroups under the FE assumption; that is, moderator effects and the within-study sampling variance are the only two sources of the variation in study effect sizes. Denote the true effect size in the *k*^th^ study by *δ*_*k*_, and denote the observed effect size in the *k*^th^ study by *d*_*k*_.^4^ Under the FE assumption, the observed effect size is given by

1 $$ d_{k} = \delta_{k} + \epsilon_{k} ={\upbeta}_{0}+{\upbeta}_{1} x_{1k}+{\upbeta}_{2} x_{2k}+ ... +{\upbeta}_{M} x_{Mk} + \epsilon_{k}, $$

where *x*_*mk*_ (*m* = 1,...,*M*) specify the values on the *M* moderators of the *k*^th^ study, and the βs are the corresponding coefficients. The sampling error *𝜖*_*k*_ is assumed to be distributed as $\mathcal {N}(0, \sigma ^{2}_{\epsilon _{k}})$, where $\sigma ^{2}_{\epsilon _{k}}$ is the sampling variance.

The sampling variance $\sigma ^{2}_{\epsilon _{k}}$ depends on the within-study sample sizes and the type of effect size. Using Hedges’ *g* as the measure of effect size, the sampling variance of the *k*^th^ study is estimated by

2 $$  \hat{\sigma}^{2}_{\epsilon_{k}}= \frac{{n_{k}^{T}}+{n_{k}^{C}}}{{n_{k}^{T}} {n_{k}^{C}}}+ \frac{{d_{k}^{2}}}{2({n_{k}^{T}}+{n_{k}^{C}})}, $$

where ${n_{k}^{T}}$, ${n_{k}^{C}}$ are the treatment and control group sample sizes of the *k* th study, respectively (Hedges and Olkin, 1985, p. 86).

The summary effect size is computed as the weighted mean, with weights $w_{k} = 1/\sigma ^{2}_{\epsilon _{k}}$:

3 $$  d_{+} = \frac {\sum d_{k}/\sigma^{2}_{\epsilon_{k}} }{\sum 1/\sigma^{2}_{\epsilon_{k}}} . $$

The measure of heterogeneity, the *Q*-statistic, is given by

4 $$  Q = {\sum}_{k=1}^{K} \frac{(d_{k} - d_{+})^{2}}{\sigma^{2}_{\epsilon_{k}}}. $$

Starting from one group including all the studies (i.e., the root node), FE meta-CART partitions the root node into two subgroups (i.e., offspring nodes), by searching through all possible splits and finds the moderator with corresponding split point that maximizes the between-subgroups heterogeneity. Denote the summary effect size of the *t*^th^ subgroup (i.e., *t*^th^ node in the tree) by *d*_*t*+_, the between-subgroups heterogeneity measure is computed as

5 $$  Q_{B} = {\sum}_{t}^{|T|} {\sum}_{k \in t} \frac{(d_{t+} - d_{++})^{2}}{\sigma^{2}_{\epsilon_{k}}}, $$

where |*T*| is the total number of subgroups^5^, and *d*_++_ is the weighted grand mean of the parent node.

**Fig. 2:**
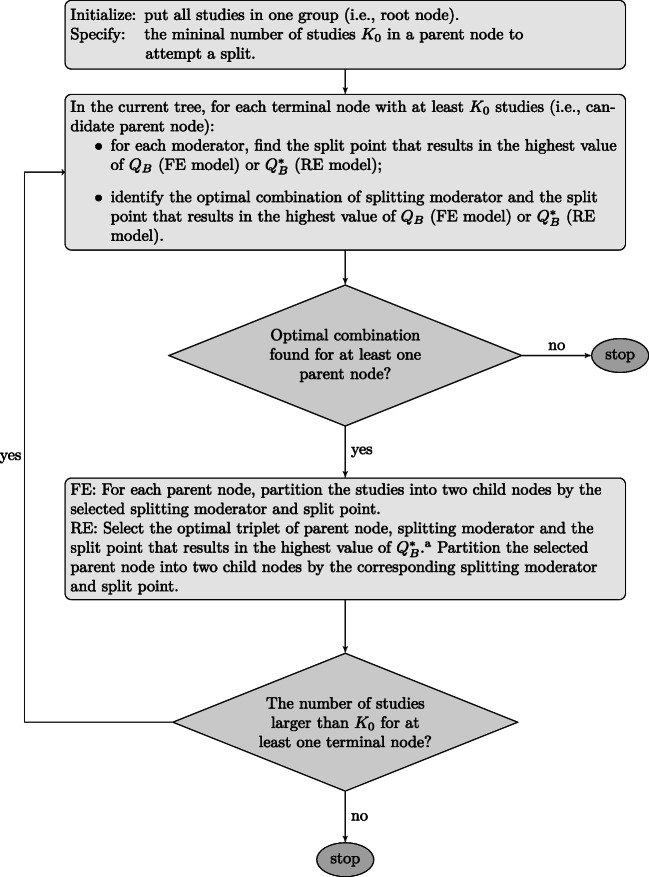
Flowchart of tree growing process of meta-CART algorithms for fixed-effect (FE) model and random-effects (RE) model. ^a^ Note that this substep is not needed for recursive partitioning method like ordinary CART and FE meta-CART, but necessary for RE meta-CART due to the re-estimation of $\sigma ^{2}_{\tau }$

To grow a tree, FE meta-CART recursively searches the split that maximizes the heterogeneity *Q*_*B*_ between the left and right child nodes. After each split, the algorithm partitions a parent node into two child nodes. The tree-growing process is a recursive partitioning procedure since the same operation can be applied to any child node itself without taking the other nodes in the current tree into account. A flowchart of the tree growing process of FE meta-CART is shown in Fig. 2. The splitting process continues until all terminal nodes contain fewer than *K*_0_ studies, where *K*_0_ is a user-specified threshold (see “The metacart package”). To prevent overfitting, the initial tree will be iteratively pruned to a nested sequence of subtrees, from which a best-sized tree is chosen via cross-validation. Different rules can be applied to determine the best-sized tree. To generalize the rules, a parameter *c* is introduced to select the “optimal” tree by using the *c* ⋅ *SE* rule (Dusseldorp et al., 2010). The *c* ⋅ *SE* rule selects the smallest tree with cross-validation error within the minimum cross-validation error plus its standard error multiplied by *c*.

The final analysis results consist of the selected tree model that represents the interactions between moderators, the estimates of the summary effect sizes *d*_*t*+_ within each subgroup, and the between-subgroups *Q*_*B*_ with *df* = |*T*|− 1. It should be noted that the significance test based on the between- subgroups *Q*-statistic is an overoptimistic pseudo *Q*-test. Given pre-defined subgroup membership, the between-subgroups *Q*-statistic follows a Chi-square distribution with *df* = |*T*|− 1 under the null hypothesis that there is no significant heterogeneity between the subgroups. However, in meta-CART the subgroup membership is not pre-defined, but identified by the tree-growing and cross-validation procedures. Therefore, over-optimism exists in the *Q*-test, and the *p* value should not be interpreted as the face value. It only gives information when the moderator effects are not significant. In other words, a non-significant *p* value indicates that the influence of the identified moderator(s) on study effect sizes is not significant, but a significant *p* value does not confirm the significance of the moderator effects. It is also worth to note that despite the over-optimism in the *Q*-test, the type I error rate of meta-CART (i.e., defined as the rate of finding a nontrivial tree with a significant between-subgroups *Q*-statistic while there is no moderator effect in the true structure underlying the data) is not inflated, because the type I error of moderator effects is mainly controlled by the pruning procedure (for details, see Li et al.,, 2019).

#### RE meta-CART

RE meta-CART takes the residual heterogeneity unexplained by moderators into account. The RE model is expressed as below:

6 $$  d_{k} = {\upbeta}_{0}+{\upbeta}_{1} x_{1k}+{\upbeta}_{2} x_{2k}+ ... +{\upbeta}_{M}x_{Mk} + \tau_{k} + \epsilon_{k}, $$

where *τ*_*k*_ is the variation introduced by the residual heterogeneity. Denote the residual heterogeneity by $\sigma ^{2}_{\tau }$, with *τ*_*k*_ distributed as $\mathcal {N}(0, \sigma ^{2}_{\tau })$. Note that there are two random components in the RE model: *τ*_*k*_ and *𝜖*_*k*_, where *τ*_*k*_ is the difference between the true effect size of the *k* th study and the mean of the population from which all study effect sizes are sampled, and *𝜖*_*k*_ is the sampling error of the observed effect size *d*_*k*_ as an estimate of the true effect size of the *k* th study (Hedges & Vevea, 1998). These two sources of variance are estimated in a hierarchical manner (Erez et al., 1996). The first level accounts for the within-study variation (or the sampling variance) $\sigma ^{2}_{\epsilon _{k}}$, which can be estimated as [2]. The second level accounts for the between-study variance (or the residual heterogeneity) $\sigma ^{2}_{\tau }$. There are various estimators for $\sigma ^{2}_{\tau }$, including the Hunter–Schmidt estimator (Schmidt & Hunter, 2014), the Hedges estimator (Hedges & Olkin, 1985), the DerSimonian–Laird estimator (DerSimonian & Laird, 1986), the Sidik–Jonkman estimator (Sidik & Jonkman, 2005), and the maximum-likelihood or restricted maximum-likelihood estimator (Viechtbauer, 2005). In the **metacart** package, we use the DerSimonian–Laird estimator for its lower computational cost.^6^

With the estimated residual heterogeneity, the summary effect size can be computed with the RE weights $w_{k}^{*} = 1/(\sigma ^{2}_{\epsilon _{k}} + \sigma ^{2}_{\tau })$:

7 $$  d_{+}^{*} = \frac {\sum d_{k}/(\sigma^{2}_{\epsilon_{k}} + \sigma^{2}_{\tau}) }{\sum 1/(\sigma^{2}_{\epsilon_{k}} + \sigma^{2}_{\tau})} . $$

The RE heterogeneity is given by

8 $$  Q^{*} = {\sum}_{k=1}^{K} \frac{(d_{k} - d^{*}_{+})^{2}}{\sigma^{2}_{\epsilon_{k}}+\sigma_{\tau}^{2}}. $$

Similar to FE meta-CART, RE meta-CART starts from the root node, and searches for the split that maximizes the between-subgroups heterogeneity. The difference is that before searching for the split, RE meta-CART needs to estimate the residual heterogeneity ($\sigma ^{2}_{\tau }$) first, since the RE between-subgroups heterogeneity is given by

9 $$  Q^{*}_{B} = {\sum}_{t}^{|T|} {\sum}_{k \in t} \frac{(d^{*}_{t+} - d^{*}_{++})^{2}}{\sigma^{2}_{\epsilon_{k}}+ \sigma_{\tau}^{2}}. $$

To continue the splitting process, RE meta-CART updates the estimate for $\sigma ^{2}_{\tau }$ and searches for the new split maximizing the partitioning criterion. A flowchart of the tree-growing process of RE meta-CART is also given in Fig. 2. Note that a split of a node will globally affect the estimation of $\sigma _{\tau }^{2}$ and the value of $Q_{B}^{*}$. Thus, in contrast to FE meta-CART, RE meta-CART considers heterogeneity between all the terminal nodes rather than only between the resulting left and right child nodes after a split. As a result, the tree-growing process of RE meta-CART is not fully recursive, since the algorithm needs to take all terminal nodes of the current tree into account to introduce a new split. In other words, the estimate of $\sigma _{\tau }^{2}$ and the optimal choice for a new split depend on the sequence of previous splits. Instead, RE meta-CART applies a sequential partitioning algorithm. 7

As in FE meta-CART, the splitting process of RE meta-CART continues until a large tree is grown. Then, an optimally sized subtree is selected using cross-validation with the *c* ⋅ *SE* rule. The associated between-subgroups $Q^{*}_{B}$, the estimates for residual heterogeneity $\sigma _{\tau }^{2}$, and the within-subgroup summary effect sizes $d^{*}_{j+}$ are obtained as the final tree is selected.

#### Look-ahead strategy for RE meta-CART

The growing algorithms of both FE and RE meta-CART are fully greedy. Although the approach guarantees a locally optimal solution at each split, it does not guarantee a globally optimal solution for the whole tree. That is, the tree-growing procedure chooses each optimal split with no regard of future splits. Because the estimated residual heterogeneity is influenced by the sequence of partitioning, the RE meta-CART is more sensitive to this local optimization problem. To alleviate this problem, we propose a look-ahead strategy for RE meta-CART, which examines two steps ahead instead of one at the split of the root node. This look-ahead strategy is applied only to the top level of a tree.

Starting the algorithm at the root node, a standard RE meta-tree chooses the single split (i.e., one split point on one moderator) that maximizes the between-subgroups heterogeneity. In contrast, a look-ahead strategy searches for the combination of two splits (i.e., single split points on two moderators or two split points on one moderator) that maximizes the partitioning criterion. As a result, the entire growing procedure that applies a look-ahead strategy consists of two sub-procedures. The first sub-procedure grows a tree with two splits, which searches for the optimal combination of a split of the root node and a split of one of its child nodes. The combination that maximizes $Q^{*}_{B}$ is chosen. In the second sub-procedure, the resulting offspring nodes of the first two splits are split following a fully greedy procedure for maximizing $Q^{*}_{B}$ at each split. This complete splitting procedure grows a large tree, which will be pruned using the pruning procedure mentioned above.

It should be noted that such a strategy does not guarantee a globally optimal solution, and only partially addresses the local optimization issue. Although more steps to look ahead can be beneficial, we chose the number of steps as two because the computational burden of a look-ahead procedure grows exponentially as the number of steps increases (Esmeir & Markovitch, 2007).

## The metacart package

The **metacart** package provides functions to perform both FE and RE meta-CART analysis. The package is available via the Comprehensive R Archive Network (CRAN) at https://cran.r-project.org/package=metacart, and can be directly installed within R by typing install.packages("metacart"). The current version is 2.0-0.

### Functions for FE meta-CART

The function FEmrt is the main function to perform a fixed effect meta-CART analysis. Both the tree growing and pruning processes in the function FEmrt() are based on the function rpart() in the **rpart** package (Therneau et al., 2017), with adaptations to fit a tree model applying appropriate weights on meta-analytic data (see “Examples”) and automatically compute subgroup meta-analysis results within the function. We now describe the different arguments of the function. 
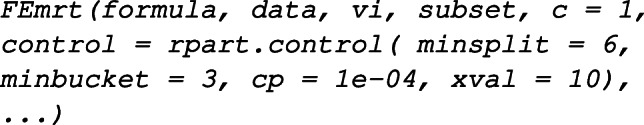

- The argument formula defines the outcome variable (i.e., effect size) and the predictor variables (i.e., moderators).
- The argument data specifies the name of the data set to be analyzed. The argument vi requires the column name of the sampling variance of the effect size.
- The argument subset is an optional expression to select a subset of the data to fit the model. The subset argument can either be a logical or a numeric vector indicating the indices of rows (i.e., studies) to be included.
- The argument c refers to the pruning parameter (described in “Fixed effect meta-CART”) to be used for the analysis. The default value c = 1 corresponds to the one-standard-error rule recommended by Breiman et al., (1985). For meta-CART analysis, the recommended value of *c* depends on the type of research at hand and the number of studies. In general, if a strict control of the type I error (less than or equal to 0.05) is required, a pruning rule using *c* = 1 can be applied when the number of studies *K* < 80, and *c* = 0.5 when *K* ≥ 80 (for more guidelines about the value of the pruning parameter *c*, (see Li et al., 2019).
- The argument control specifies the options to control the growing and pruning processes. As mentioned above, both the tree growing and pruning processes are based on the function rpart, and therefore the control argument should be an rpart.control object (see details in Thernau et al., 2017). Within the rpart.control object, minsplit specifies the minimum number of studies that must exist in a parent node for a split to be attempted; minbucket specifies the minimum number of studies in any terminal node; cp specifies the minimal improve of complexity parameter (i.e., *Q*_*B*_ divided by *Q*) to make a split in the growing process; xval specifies the number of cross-validations in the pruning process. The default value of minbucket is chosen as 3, because a sample size of one or two is too small to produce reliable subgroup meta-analysis results. Consequently, the default value of minsplit is chosen as 6. The default value xval= 10 corresponds to the ten-fold cross-validation recommended by Breiman et al., (1985).

The output of the FEmrt function is an S3 object of class "FEmrt". The corresponding print and summary methods can be used to display and inspect the elements of the object. The plot method can be used to present the main effects of identified moderators and interaction effects between them in a tree model. The predict method of the S3 object allows the user to predict effect size given the value of moderators. Examples to apply these methods will be described in “Examples”.

### Functions for RE meta-CART

The function REmrt is the main function to perform random effects meta-CART analysis. Both the tree growing and pruning processes in the function REmrt are entirely new **R**-code combined with **C++** that implements the sequential partitioning algorithm described in “RE meta-CART”. The different arguments of the function REmrt are described below. 
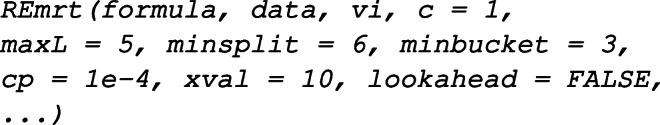

- The arguments formula, data, vi, and c are similar to the corresponding arguments in the function FEmrt.
- The argument maxL is an option to define the maximum number of splits in the tree growing process.
- The arguments minsplit, minbucket, cp, and xval are other options to control the growing and pruning processes. These options are not given in an rpart.control object, because the REmrt is an entirely new function and does not depend on the rpart function. The specifications of these options are the same as those from the function FEmrt.
- The argument lookahead is a logical indicator to specify whether to apply the look-ahead strategy described in “Examples”.

The output of the REmrt function is an S3 object of class "REmrt". Similar to FEmrt, there are corresponding print, summary, plot, and predict methods for "REmrt" objects. Examples will be described in “Examples”.

## Examples

### New approach meta-CART compared to meta-regression

In this example, we re-analyze the data "dat.bourassa1996" included in the **metafor** package (Viechtbauer, 2010). The data set contains the meta-analytic data from Bourassa et al., (1996), including results from 47 studies on the association between handedness and eye-dominance. Some studies included multiple (independent) samples, resulting in 54 samples in total. Furthermore, for some studies, the combined data of the males and females were further broken down into the two subgroups. As a result, the data set contains 96 (sub)samples in total. We only selected the independent samples with the combined data of both males and females (*K* = 54). The results of each of these samples were given in terms of the number of left-handed left-eyed, left-handed right-eyed, right-handed left-eyed, and right-handed right-eyed individuals. We use the log odds-ratio as the measure of effect size, which is the same as in Bourassa et al., (1996). A higher log odds-ratio indicates a higher association between handedness and eye dominance. First, we compute the effect size and sampling variance by using the escalc function in the **metafor** package. 
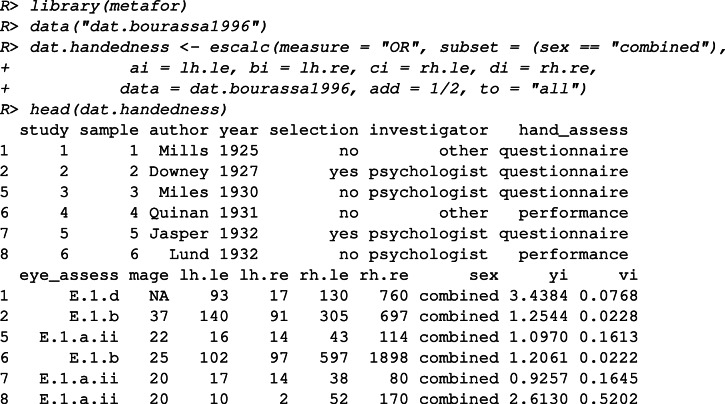


The computed effect size ("yi") and variance ("vi") are added to the data set. The data set provides information about the following moderators: the publication year ("year"), whether the selection of subjects was based on eye-dominance or handedness ("selection", with two categories), the type of investigator ("investigator", with three categories^8^), the method to assess handedness ("hand_assess", with two categories), the methods to assess eye-dominance ("eye_assess", with six categories), the average age ("mage").

Both meta-regression and meta-CART are applied to re-analyze this data set. The analyses results are compared to illustrate the different research questions that can be answered by these approaches. For both analyses, the moderators “selection”, “investigator”, “hand_assess”, and “eye_assess” are selected. The random effects model is chosen and the DerSimonian–Laird estimator is used to estimate the residual heterogeneity. We use “set.seed(2018)” so that the analysis results described in this paper can be replicated.

First, to answer the question “which moderators affect hand-eye association?”, we fit a meta-regression model with the main effects of the selected moderators. 
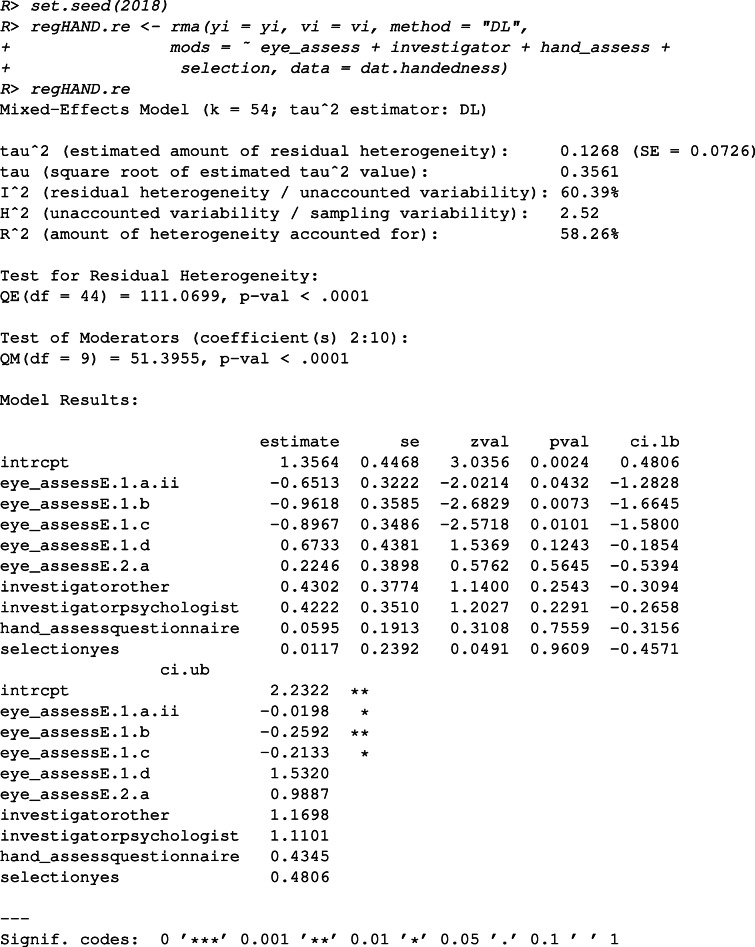


The output shows that three contrasts derived from the method to assess eye-dominance are significant: contrasted with the eye-dominance assessment method “E.1.a.i” (i.e., the reference method), “E.1.a.ii”, “E.1.b”, and “E.1.c” result in significantly lower hand-eye association. The remaining two methods, “E.2.a” and “E.1.d” do not differ significantly from the reference method. Note that the interpretation of these contrasts depends highly on the choice of reference method.

Further analyses are needed if we are interested in whether some categories of a moderator can be combined and whether interaction effects between the moderators are present. Alternatively, meta-CART can be performed to answer the question “which combinations of (categories of) moderators are influential?”. The combinations can be either combinations of multiple moderators, or combinations of multiple categories of one moderator. We fit a RE meta-CART model by 
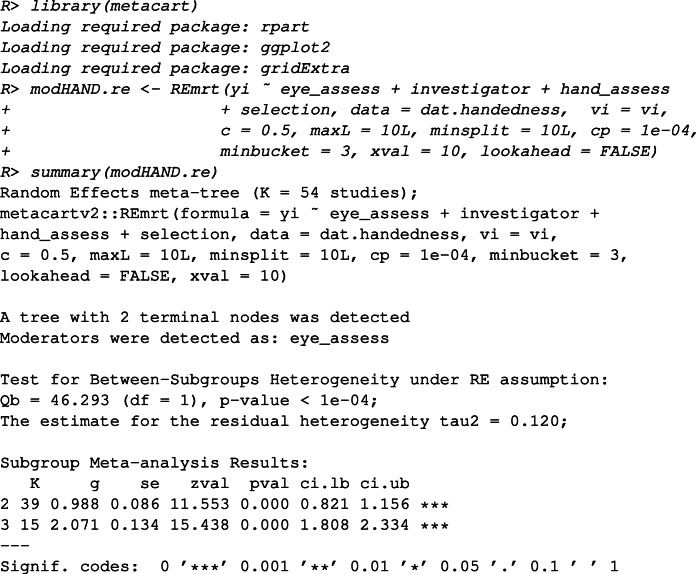
 The plot function can be used to inspect the influence of eye-dominance assessment methods on the association between handedness and eye-dominance. 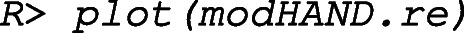 The plot can be found in Fig. 1, which shows a tree with one split and two terminal nodes. Note that this tree results from pruning an initially large tree based on cross-validation (see 4). According to the summary results and the plot, the moderator “eye_assess” has a strong influence on the association between handedness and eye-dominance ($Q^{*}_{B} = 46.29$, *df* = 1, *p* value < 0.0001). When the eye-dominance is assessed using the methods “E.1.a.ii”, “E.1.b”, or “E.1.c”, the observed association between handedness and eye-dominance is lower (log odds-ratio = 0.988, 95*%* CI: 0.821 to 1.156). When the eye-dominance is assessed using the methods “E.1.a.i”, “E.1.d”, or “E.2.a”, the observed association is generally higher (log odds-ratio = 2.071, 95*%* CI: 1.808 to 2.334). This is in accordance with the claims in Bourassa et al., (1996) that the methods to assess eye-dominance can be partitioned into two subgroups: (1) unbiased methods including “E.1.a.ii: monocular procedure with object/instrument held in both hands”, “E.1.b: binocular procedure”, and “E.1.c: a combination of the previous methods”, (2) biased methods including “E.1.a.i: monocular procedure with object/instrument held in one hand”, “E.2.a: assessment based on a questionnaire”, and “E.1.d: some other method”. The methods “E.1.a.ii”, “E.1.b”, or “E.1.c” were symmetric in the sense that both hands are used equivalently during the process of measurement, and therefore are less likely to have measurement bias.

Comparing to meta-regression, which estimates the coefficients and tests the significance for the contrasts derived from “eye_assess”, meta-CART partitions the multi-categorical moderator “eye_assess” into subgroups, and tests the heterogeneity between the resulting subgroups. The subgrouping membership can be used to verify prior hypotheses^9^, or to generate hypotheses if no a priori hypotheses exist.

### Identify interaction effects using FE/RE assumptions

In this example, we perform a meta-analysis to identify the most effective combination of treatment components by exploring the interactions between them. The **metacart** package provides the data object dat.BCT2009 as a subset of the meta-analytic data from Michie et al., (2009). The meta-analysis by Michie et al., (2009) aimed to assess the effectiveness of interventions designed to promote physical activity and health eating, and investigated whether theoretically specified behavior change techniques (BCTs) improve the effectiveness. The subset used in this example consists of 106 interventions that included at least one of the motivation-enhancing BCTs. 
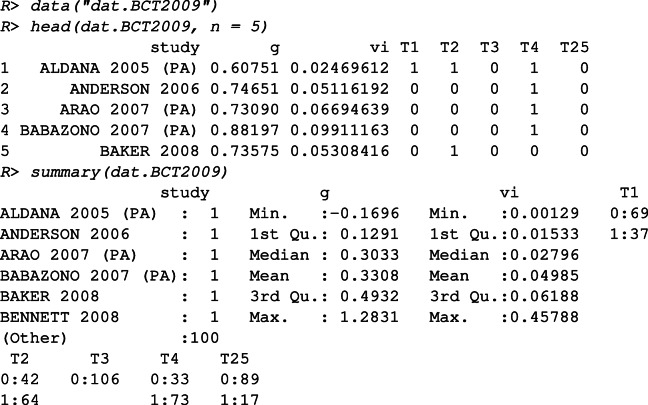
 As displayed above, the data set contains information about the name and the publication year, the estimated effect size (*g* = Hedges’*g*, which denotes the standardized mean difference in treatment outcome), the sampling variance of the effect size, and whether specific BCTs were applied or not in a study (“0” for absent and “1” for present). In this example, we will re-analyze this data set focusing on the motivation-enhancing BCTs that may explain the heterogeneity in the effect sizes of the interventions. We use the pruning parameter *c* = 0.5 for both FE and RE meta-CART analyses. Four moderators “T1: Provide information about behavior-health link”, “T2: Provide information on consequences”, “T4: Prompt intention formation”, and “T25: Motivational interviewing” are included in the meta-CART analysis. The moderator “T3: Provide information about other’s approval” is excluded since none of the studies applied this BCT.

If we have prior knowledge that the FE assumption is reasonable here, a FE meta-CART model can be fitted using the following code 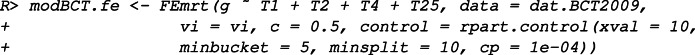


**Fig. 3:**
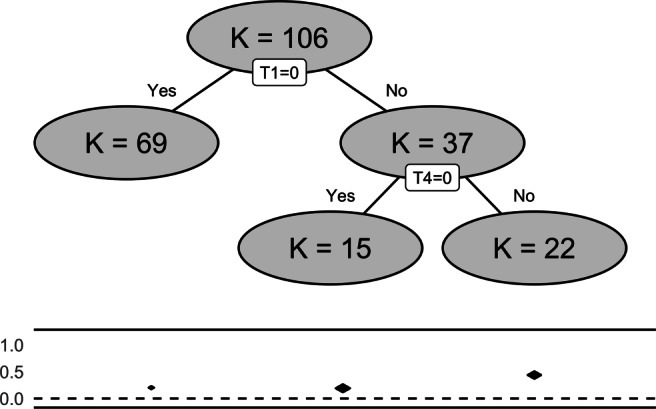
The meta-CART analysis result of 106 studies that examine the influence of motivation-enhancing BCTs on healthy eating and physical activities. The figure shows the FE meta-CART structure with splitting information at each internal node and the number of studies in each subgroup implied by a terminal node. The *two solid lines* show the range of the effect sizes of all the studies. The *diamonds* between the solid lines present the 95% confidence intervals of the summary effect sizes.

To obtain the summary output, we use 
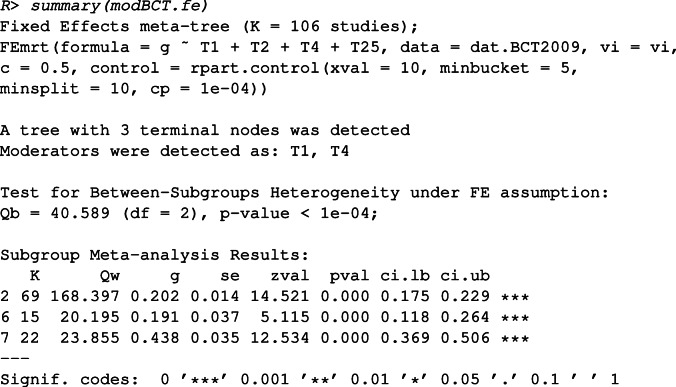
 This output shows that a tree with three terminal nodes was detected. The studies are partitioned into three subgroups based on two influential moderators “T1” and “T4”. The between-subgroups heterogeneity is significant (*Q*_*B*_ = 40.589, *df* = 2, *p* value < 0.0001). The subgroup analysis results show the number of studies (*K*), the within-subgroup *Q*-statistic, the summary effect sizes (*g*) in each subgroup, the standard errors of the summary effect sizes (se), *Z*-test statistics of the summary effect sizes, and the confidence intervals of the summary effect sizes. The plot function can be used to inspect the final tree, in this case, the interaction between the two moderators “T1” and “T4”. The plot is shown in Fig. 3. If an intervention does not include “T1”(i.e., *T*_1_ = 0 is true), then the intervention ends up in the left terminal node (with *K* = 69). For those that include “T1” but not include “T4”, they end up in the middle terminal node (with *K* = 15). Interventions including both “T1” and “T4” end up in the right terminal node (with *K* = 22). Combined with the other results, we can see that the summary effect size is the highest when ”T1” and ”T4” are both present in the intervention (*g* = 0.438, 95*%* CI: 0.369 to 0.506).

If a new intervention is designed with three BCTs “T1”, “T2” and “T25” (thus without “T4”), the prediction of its effect size based on this tree (modBCT.fe) can be obtained by the predict function. 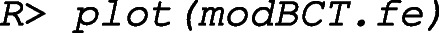

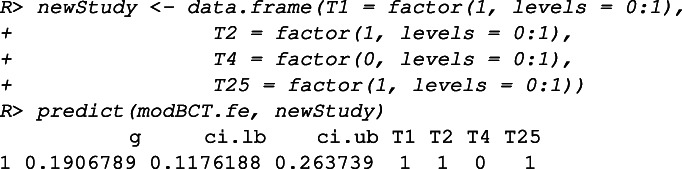
 When interpreting the FE meta-CART analysis results, it is important to realize that the FE assumption ignores the uncertainty introduced by the residual heterogeneity. As a result, the confidence intervals of the summary effect sizes are more narrow than those estimated by using the RE model.

If we would like to take into account the residual heterogeneity, a RE meta-CART model can be fitted using the following commands 
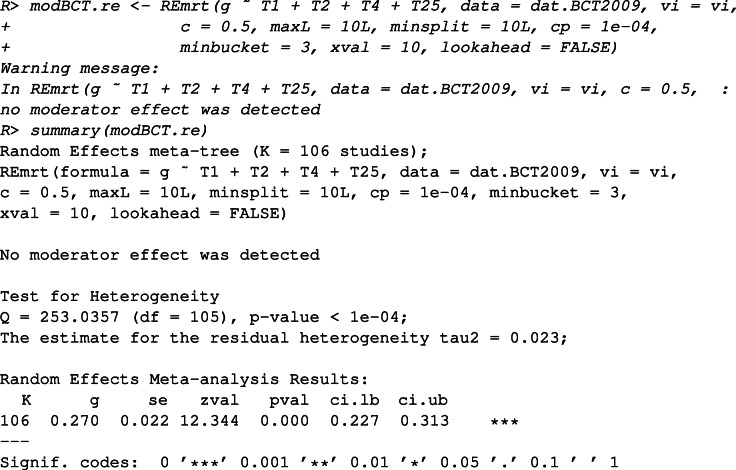
 The message “no moderator effect was detected” indicates that the cross-validation procedure selected the best-sized subtree with no splits, which means that no influential moderators were identified. In this case, the summary method shows the standard RE meta-analysis results instead of the subgroup analysis results. The heterogeneity among the studies is significant ($Q_{B}^{*} = 253.036$, *df* = 105, *p* value < 0.0001). The estimated summary effect size for all studies (*K* = 106) is 0.270 (95*%* CI: 0.227 to 0.313).

### Look-ahead strategy

The **metacart** package provides a simulated data set dat.balanced to illustrate the look-ahead strategy for RE meta-CART analysis. The simulated data set contains *K* = 60 studies with four moderators: *x*_1_, *x*_2_, *x*_3_, *x*_4_, among which *x*_1_, *x*_2_, and *x*_4_ are randomly sampled dichotomous variables and *x*_3_ is sampled from a uniform distribution *U*(0,1). The sample size *n* was generated from a normal distribution $\mathcal {N}(160, (\frac {160}{3})^{2}$). The residual heterogeneity was set as $\sigma _{\tau }^{2} = 0.01$. The true model used to generate data was

10 $$  d_{k} = 0.5 \cdot I(x_{1} = 0, x_{2} = 1) + 0.5 \cdot I(x_{2} = 0, x_{1} = 1) + \tau_{k} + \epsilon_{k}. $$

This model is similar to the simulated example used by Tibshirani and Knight (1999). This model is shown by Tibshirani and Knight (1999) to be difficult for greedy search procedures like CART because there is no information on where to split at the top level. Due to the same reason, standard meta-CART is likely to end up with a local optimum solution in such case. In this example, we would like to show that the look-ahead strategy described in “Examples” can alleviate this problem.

First, we inspect the data and fit a RE meta-CART model without using the look-ahead strategy. 
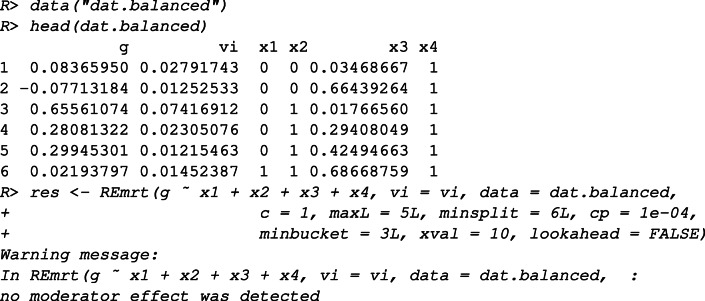
 The output shows that meta-CART failed to detect any moderator. The reason is that the algorithm got stuck at a local optimum in the splitting procedure. This can be verified by inspecting the initial tree, unpruned by cross-validation. 
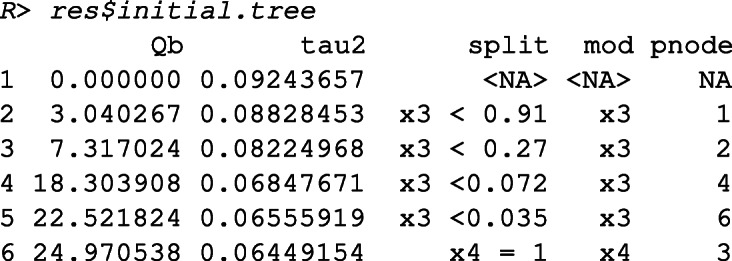
 This output shows the between-subgroups *Q*-statistic, the residual heterogeneity, the chosen split points (by default the values are shown in two decimal places), the chosen moderator, and the parent node to be partitioned at each split, with the first row presenting the root node when no split occurs. From the output we can see that the algorithm falsely chose the first splits at *x*_3_ and ended up with *Q*_*B*_ = 24.97 afterfive splits.

Then we fit a RE meta-CART model using the look-ahead strategy. 
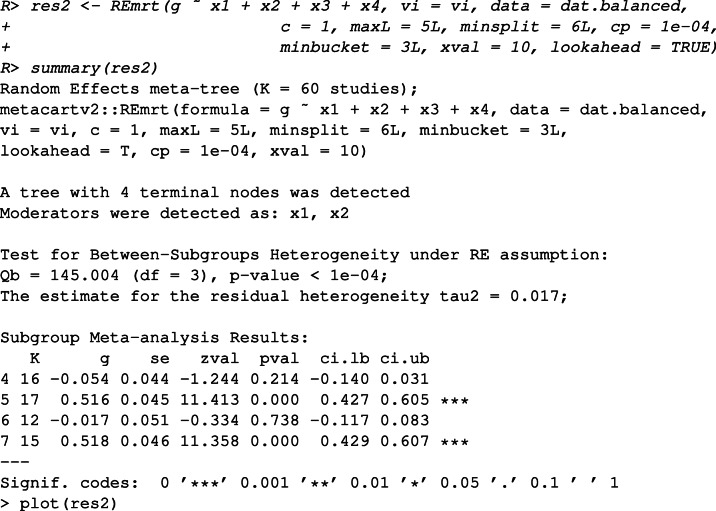


**Fig. 4:**
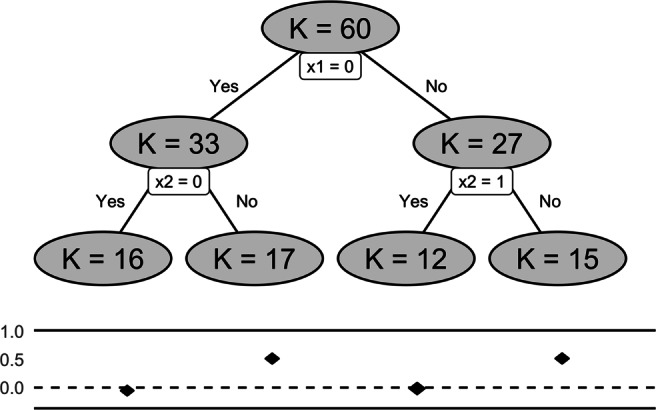
The analysis results of simulated data generated by fitting a RE meta-CART model with look-ahead strategy. The analysis successfully recovered the true model that was used to generate the simulated data

The results show that the RE meta-CART model with look-ahead strategy successfully recovered the true model as in (10). A tree with four terminal nodes was detected. As shown in Fig. 4, the terminal node with *x*_1_ = 0 and *x*_2_ = 1 (*K* = 17) and the terminal node with *x*_1_ = 1 and *x*_2_ = 0 (*K* = 15) have higher effect sizes with CIs (presented by the diamonds) covering 0.5, whereas the terminal node with *x*_1_ and *x*_2_ both equal to 0 (*K* = 16) and the terminal node with *x*_1_ and *x*_2_ both equal to 1 (*K* = 12) have lower effect sizes with CIs covering 0.

The initial tree obtained with look-ahead strategy can be inspected by 
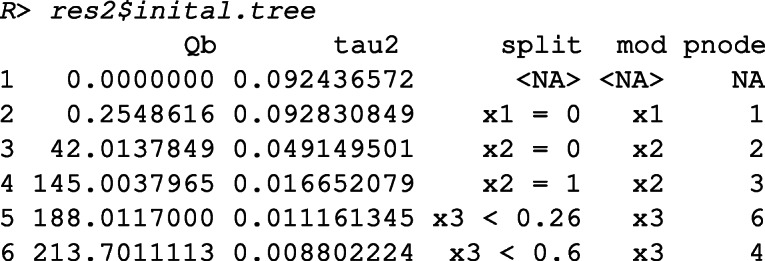
 Comparing this tree to the initial tree obtained from the previous model without the look-ahead strategy, the look-ahead strategy correctly finds the first two splits, and obtains a solution with much larger between-subgroups *Q*-statistic (*Q*_*B*_ = 213.70 after five splits).

## Conclusions

This paper presents the **metacart** package written in R to perform meta-CART analysis both for fixed effect and random effects models. The algorithms and the main functions of the package **metacart** are described in “Meta-CART method” and “The metacart package”, respectively. Applications of the **R**-package **metacart** were illustrated through example analyses on two real-world data sets and one simulated data set in “Examples”.

One strength of the package **metacart** is that it can easily explore the interaction effects among multiple moderators using an interpretable tree model. Furthermore, for multi-categorical variables, it creates automatically the contrasts between (combinations of) categories that account for the highest amount of heterogeneity. Another strength is that **metacart** provides researchers various options for the tree growing and pruning processes. For example, researchers can choose the minimum number of studies in parent nodes and the minimum number of studies in terminal nodes based on the total sample size. In general, it is recommended to have at least three studies in each terminal nodes. The pruning parameter can be chosen based on the balance between power and type I error. A detailed guideline for choosing the pruning parameter can be found in Li et al., (2019). The look-ahead strategy is recommended to partially relieve the local optimization problem when fitting a RE meta-CART model.

In conclusion, this paper and the developed **metacart** package introduce researchers to the implementation of meta-CART analysis, to facilitate exploring interaction effects between multiple moderators in the framework of meta-analysis.

## Electronic supplementary material

Below is the link to the electronic supplementary material.

(PDF 138 KB)

## Footnotes

1 Most effect-size measures can be converted to one another (see Hedges and Olkin 1985).

2 the estimated confidence intervals are normal-theory 95% CIs. The metacart package always provides 95% CIs for the estimated effect sizes. If user is interested in CIs of other confidence levels, they can be easily computed based on the standard error of the estimates.

3 This implies that differences between the studies are all accounted for by moderators, and sampling variance accounts for the variance of the study effect sizes adjusted for moderator effects.

4 Note that we focus in the description on the *d*-family of effect sizes, that is, measures of the standardized mean outcome difference between treatment and control groups (e.g., Cohen’s *d* or Hedges’ *g*). However, the package metacart can also be used for other effect size measures such as log odds ratio (see the example in “New approach meta-CART compared to meta-regression”), log relative risk, and Fisher’s *z*.

5 |*T*| = 2 when only considering the heterogeneity between left and right child nodes after a split of the parent node.

6 In our pilot simulation study, it was found that the different choices of the estimator led to similar trees.

7 A sequential tree-growing algorithm is defined as an algorithm that only splits a node in the tree if the reduction in the partitioning criterion at the proposed node exceeds some value, regardless of what might happen lower in the tree (Hand, 1997, Chapter 4). Recursive partitioning can be seen as a restricted form of sequential partitioning where at each node, exactly the same splitting procedure is repeated.

8 The three categories are “psychologist”, “educationalist”, and “other”

9 If the subgroup membership is tested as a categorical moderator, meta-regression yields the same results as meta-CART.
