# Supplementary material for: Multiple moderator meta-analysis using the R-package Meta-CART
Source: Behav Res Methods. 2020 Jun 15;52(6):2657–73. doi: 10.3758/s13428-020-01360-0 (PMC7725699; doi:10.3758/s13428-020-01360-0)
Supplement: Supplementary file 1 — (PDF 138 KB) [file 13428_2020_1360_MOESM1_ESM.pdf]

Supporting Information: residual heterogeneity in random-effects meta-CART

In random-effects meta-analysis, it is assumed that there is heterogeneity unexplained by the moderators, and such heterogeneity is called residual heterogeneity. In meta-CART analysis, we estimate the residual heterogeneity using the DerSimonian and Laird (1986) method. The residual heterogeneity is computed as

$$\sigma_\tau^2 = \frac{\sum_{j=1}^{J} Q_j - \sum_{j=1}^{J} df_j}{\sum_{j=1}^{J} C_j}, \tag{11}$$

where Q_j is the within-subgroup Q-statistic in the jth group. It can be computed as in (4). df_j equals $K - 1$, and the components C_j are computed as

$$C_j = \sum_{k=1}^{K} \frac{1}{\sigma_{\epsilon_{jk}}^2} - \frac{\sum_{k=1}^{K} 1/\sigma_{\epsilon_{jk}}^4}{\sum_{k=1}^{K} 1/\sigma_{\epsilon_{jk}}^2}. \tag{12}$$

In the tree growing process of RE meta-CART, the residual heterogeneity σ_τ^2 is updated after each split. Once a new split is introduced, the estimation of σ_τ^2 needs to be re-estimated based on the new values of Q_j, df_j and C_j. As a result, the random effects within-subgroup Q_j^* needs be re-computed for all the existing terminal nodes in the current tree.
